# Acne Inversa-like Lesions Induced by a Low Dose of Sorafenib

**DOI:** 10.5826/dpc.1102a08

**Published:** 2021-03-08

**Authors:** Javier Aubán-Pariente, Laura Palacios-García, Cristina Galache-Osuna, Marc Mir-Bonafé, Patricia Morales Del Burgo, Jorge Santos-Juanes

**Affiliations:** 1 Department of Dermatology, Central University Hospital of Asturias, Oviedo, Spain; 2 Department of Pathology, Central University Hospital of Asturias, Oviedo, Spain

**Keywords:** acne inversa, sorafenib, cutaneous adverse reactions, dermatopathology

## Introduction

Sorafenib is an oral multikinase inhibitor approved for the treatment of unresectable hepatocellular carcinoma, differentiated thyroid cancer, and advanced renal-cell carcinoma. This drug blocks tumor cell proliferation and angiogenesis by inhibiting the Raf serine/threonine kinases (*RAF1* and *BRAF*) as well as multiple receptor tyrosine kinases [1]. Adverse skin reactions occur in up to 90% of patients. Although acneiform eruptions have been widely reported in association with epidermal growth factor receptor (EGFR) inhibition, ranging from 24%–91% of patients, those induced by sorafenib are rare. They usually appear in the first 6 weeks after drug onset and are located on the face. Regarding clinical-pathological characteristics, acneiform eruptions induced by sorafenib are classified into 3 groups: papulopustular eruptions without associated retention lesions, nodular-cystic eruptions, and perforating folliculitis [2]. Of these groups, papulopustular facial eruptions have been the most frequently observed. Here we report a case of acne inversa-like lesions induced by a low dose of sorafenib in a 62-year-old man with hepatocellular carcinoma.

## Case Presentation

**Figure 1:**
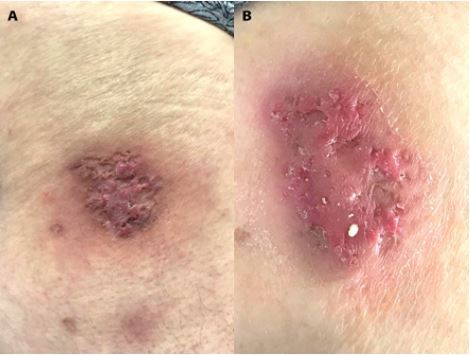
(A, B) Two indurated erythematous plaques with fistulous tracts and depressed scar areas on both legs.

A 62-year-old man was referred to us for evaluation of 2 exudative lesions on his lower extremities of 1-month duration. His medical history included sorafenib 400 mg/day for 4 months for diffuse hepatocellular carcinoma. The patient denied a history of similar lesions prior to the onset of sorafenib. Physical examination revealed 2 indurated erythematous plaques on both thighs that were 2 to 6 cm in diameter (Figure 1, A and B). The lesions had small oozing cavities, fistulous tracts, and depressed scar areas. No involvement of other locations was observed.

**Figure 2:**
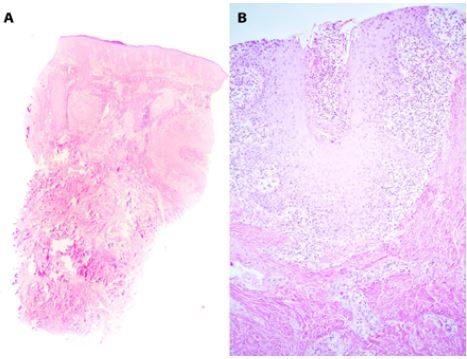
(A) Perifollicular and perivascular lymphohistiocytic infiltration (H&E, ×20). (B) Dilated follicular infundibula filled with compact parakeratotic cornified cells and neutrophils (H&E, ×100).

Skin swabs for viral and bacterial cultures were negative. A skin biopsy showed perifollicular and perivascular lymphohistiocytic infiltration (Figure 2A). Dilated follicular infundibula were filled with compact parakeratotic cornified cells and neutrophils (Figure 2B). A diagnosis of acne inversa-like lesions induced by sorafenib was made, and topic fusidic acid was prescribed twice a day. The oncologist temporarily discontinued treatment with sorafenib due to liver decompensation. After 1 month of sorafenib interruption, there was complete resolution of the eruption with mild scarring.

## Conclusions

Five cases of acne inversa-like lesions associated with sorafenib have been reported, all of them in males [1,2]. Clinical features of 4 of these patients were similar to the case presented here, and except for 1 case of classic hidradenitis suppurativa located in the bilateral axillae and inguinal region, the localization was atypical in affecting the legs, abdomen, or buttocks. Histopathological analysis showed comedone-like follicular dilatation with perifollicular lymphohistiocytic infiltration. There appears to be a dose-dependent relationship between sorafenib and acneiform reaction. Except in 1 case in which an acne inversa-like eruption resolved spontaneously, lesions only improved with dose reduction or treatment interruption in combination with topical retinoids, antibiotics, or benzoyl peroxide [1,2].

If we apply the Naranjo drug reaction assessment tool to this case, the result would be “probable.” Unlike previously reported cases, our patient developed acneiform lesions while on a low dose of 200 mg twice a day. Further studies are needed to elucidate the pathogenic explanation and dose relationship between sorafenib and acne inversa-like lesions.
